# Clathrin-Independent Entry of Baculovirus Triggers Uptake of *E. coli* in Non-Phagocytic Human Cells

**DOI:** 10.1371/journal.pone.0005093

**Published:** 2009-04-07

**Authors:** Johanna P. Laakkonen, Anna R. Mäkelä, Elina Kakkonen, Paula Turkki, Sari Kukkonen, Johan Peränen, Seppo Ylä-Herttuala, Kari J. Airenne, Christian Oker-Blom, Maija Vihinen-Ranta, Varpu Marjomäki

**Affiliations:** 1 Department of Biological and Environmental Science/Nanoscience Center, University of Jyväskylä, Jyväskylä, Finland; 2 Institute of Biotechnology, University of Helsinki, Helsinki, Finland; 3 Department of Biotechnology and Molecular Medicine, A.I. Virtanen Institute, University of Kuopio, Kuopio, Finland; The University of Queensland, Australia

## Abstract

The prototype baculovirus, *Autographa californica* multiple nucleopolyhedrovirus, an insect pathogen, holds great potential as a gene therapy vector. To develop transductional targeting and gene delivery by baculovirus, we focused on characterizing the nature and regulation of its uptake in human cancer cells. Baculovirus entered the cells along fluid-phase markers from the raft areas into smooth-surfaced vesicles devoid of clathrin. Notably, regulators associated with macropinocytosis, namely EIPA, Pak1, Rab34, and Rac1, had no significant effect on viral transduction, and the virus did not induce fluid-phase uptake. The internalization and nuclear uptake was, however, affected by mutants of RhoA, and of Arf6, a regulator of clathrin-independent entry. Furthermore, the entry of baculovirus induced ruffle formation and triggered the uptake of fluorescent *E. coli* bioparticles. To conclude, baculovirus enters human cells via a clathrin-independent pathway, which is able to trigger bacterial uptake. This study increases our understanding of virus entry strategies and gives new insight into baculovirus-mediated gene delivery in human cells.

## Introduction

The baculovirus under study, *Autographa californica* multiple nucleopolyhedrovirus, is a large, enveloped, dsDNA virus that belongs to the family of *Baculoviridae*. Baculoviruses are arthropod-specific viruses ubiquitously found in the environment, of which members have been isolated from more than 600 host insect species. They play an important ecological role in regulating the size of insect populations, and their complexity in form and function suggest a long evolutionary lineage. Most baculoviruses have been isolated from the order Lepidoptera, and Lepidopteran baculoviruses are also the best characterized [1]. Baculoviruses are unique compared to other virus families by having two distinct viral phenotypes, occlusion-derived virion and budded virion, but with a shared genotype [1], [2], [3]. The occlusion-derived virion is specialized and only infects the highly differentiated columnar epithelial cells within the alkaline conditions of the larval midgut. The budded virion, on the other hand, is generalized and highly infectious to the tissues of the host as well as cultured insect cells. Traditionally, baculoviruses have been applied as targeted biocontrol agents and for heterologous gene expression in insect cells and larvae [4]. Most of the established data on baculovirus and mammalian cell interactions and baculovirus display technology relates to the budded virion [5], [6].

Apart from efficiently infecting arthropods and cultured insect cells, baculoviruses are also capable of successfully transducing various mammalian cell types [7], [8]. The receptor(s) promoting cellular binding and subsequent uptake of the virus into insect or mammalian cells are currently unidentified. As the virus is able to enter a vast variety of cell types, abundant plasmalemmal molecules, such as heparan sulfate [9] and phospholipids [10] have been suggested to participate in the binding process. Low early endosomal pH was shown to be crucial for the release of the viral capsid, and for efficient transduction in both insect and mammalian cells [11], [12], [13], [14]. Despite early endosomal targeting and occasional viral attachment to plasma membrane-bound coated pits, no baculoviruses have been observed within budded clathrin-coated vesicles [12]. Recently, Long *et al*. [15] demonstrated inhibition of baculovirus-mediated transduction in baby hamster kidney, BHK21 cells treated with chlorpromazine, suggestive for clathrin-mediated endocytosis (CME). In contrast, we detected enveloped baculovirus in numerous large plasma membrane invaginations and non-coated vesicles associated with plasma membrane ruffling, indicating that a more efficient endocytic pathway could be involved [12].

Since the discovery that baculoviruses are able to transduce cells of mammalian origin this viral vector system has been used in versatile applications in biomedicine, including vaccination as well as in cancer and immunotherapy [16], [17], [18]. So far, baculovirus vectors have been used for *in vivo* applications in animal models including rabbits, mice and rats [6]. In recent years, the transduction efficiency and the method of delivery have been optimized in serum-free environments, leading to successful transduction of neural cells [19], carotid arteries [20] and ocular tissue [21], for example. In this study, we focused on elucidating the nature of baculovirus entry in human cells. We show that the functional entry of baculovirus occurs via clathrin-independent large smooth-surfaced vesicles and that it induces the uptake of *E. coli* in non-phagocytic human cells.

## Results

### Clathrin-mediated endocytosis is not required for baculovirus transduction

**Figure 1:**
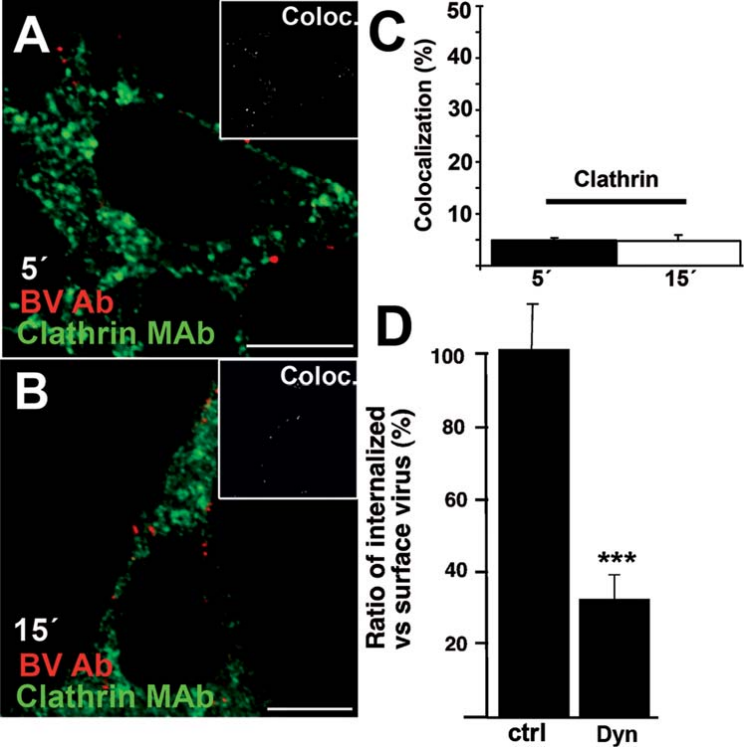
CME is not involved in baculovirus entry. 293 cells that were allowed to internalize baculovirus (wt) for 5 (A) and 15 min (B) were immunolabeled for baculovirus (red) and clathrin heavy chain (green). Colocalized voxels are shown in the inset. Scale bar, 10 µm. (C) Colocalization between baculovirus and clathrin heavy chain in 293 cells were calculated from confocal sections using the colocalization algorithm in the BioimageXD software (See Materials and Methods) from 3 separate samples, from 30 cells. Mean values and standard deviations are shown. (D) Differential labeling of intracellular (baculovirus Ab, Alexa488) and surface-bound baculovirus (baculovirus Ab, Alexa555) at 30 min was measured in dynasore-treated (Dyn, 80 µM) HepG2 cells from three separate samples (30 cells). Confocal sections were further analyzed by BioimageXD using the internalization algorithm. Mean values and standard deviations are shown.

To obtain evidence as to whether or not CME is employed by the baculovirus in mammalian cells, we used various approaches; e.g. marker proteins, small interfering RNA (siRNA), chemical inhibitors, and transfection of plasmids encoding dominant negative (DN) and constitutively active (CA) protein factors. First, the baculovirus was cointernalized or consecutively fed with Alexa546-labeled transferrin (A546-TF), the endocytic marker for the clathrin-mediated pathway, in 293 and HepG2 cells (5–60 min) and observed by confocal microscopy. In both cell types, A546-TF was efficiently internalized to cells, whereas the viral uptake appeared to progress at a slower rate. No colocalization of A546-TF with baculovirus was detected, suggesting that the baculovirus did not enter along with transferrin and was not further directed to the recycling pathway (Figure S1E). Further, the clathrin light chain tomato fusion protein was expressed in 293 cells to examine if baculovirus entry affected the cellular distribution of clathrin. Live confocal microscopy revealed that the distribution of the expressed clathrin light chain was not altered in the presence of baculovirus (Figure S1A, S1B). After 1 h post transduction (p.t.), baculovirus was internalized into large, distinct vesicles in contrast to clathrin, which was localized in small vesicles present throughout the cytoplasm. Additionally, hardly any colocalization between the virus and clathrin heavy chain antibody was detected at 5–15 min p.t. in 293 cells by confocal microscopy (Figure 1A, 1B, and 1C) or in clathrin coated vesicles by EM (MOIs 500–1000; data not shown). We also tested the effect of chlorpromazine for baculovirus uptake during 30 min of internalization. The results showed no statistically significant effect on baculovirus entry in chlorpromazine treated cells (control uptake set to 100%±14% SE compared to 83%±9% SE; p = 0.17 by one-tail t-test).

To test the involvement of dynamin in baculovirus uptake, a small molecule called dynasore was used. Dynasore is an inhibitor of dynamins, which blocks the formation of clathrin coated vesicles [22]. Dynamin is also involved in various other entry routes (e.g. caveolae-pathway, clathrin-independent IL2-receptor pathway and phagocytosis [23]). In these experiments, internalization of A546-TF was used to control of the functionality of the drug. The entry of transferrin into live 293 cells treated with dynasore was efficiently inhibited (internalization level dropped to 4.3%±1.5% SD; Figure S1F) as compared to untreated control cells (100%). Immunolabeling of the baculoviruses before and after cellular permeabilization in the presence and absence of dynasore was performed to differentiate the virus outside and inside the cell (Figure 1D). The extent of viral internalization was quantified using the colocalization and internalization algorithms embedded in the BioimageXD software [24]. The results showed that dynasore caused a 67% (*P* = 0.0005) reduction in baculovirus internalization. In line with these results, only weak baculovirus-mediated expression of the EGFP reporter protein was observed in dynasore-treated 293 cells (data not shown). The use of DN dynamin (K44A) and siRNA against dynamin-2 in 293 cells did not result in sufficient inhibition of transferrin entry, which is used as a proof of their effect, and therefore reliable baculovirus uptake studies could not be performed using these approaches (data not shown). Overall, we can conlude that the results obtained with dynasore suggest that the virions enter human cells via dynamin-dependent means, however, the results are merely indicative and not conclusive.

### Baculovirus internalizes along with fluid-phase markers

**Figure 2:**
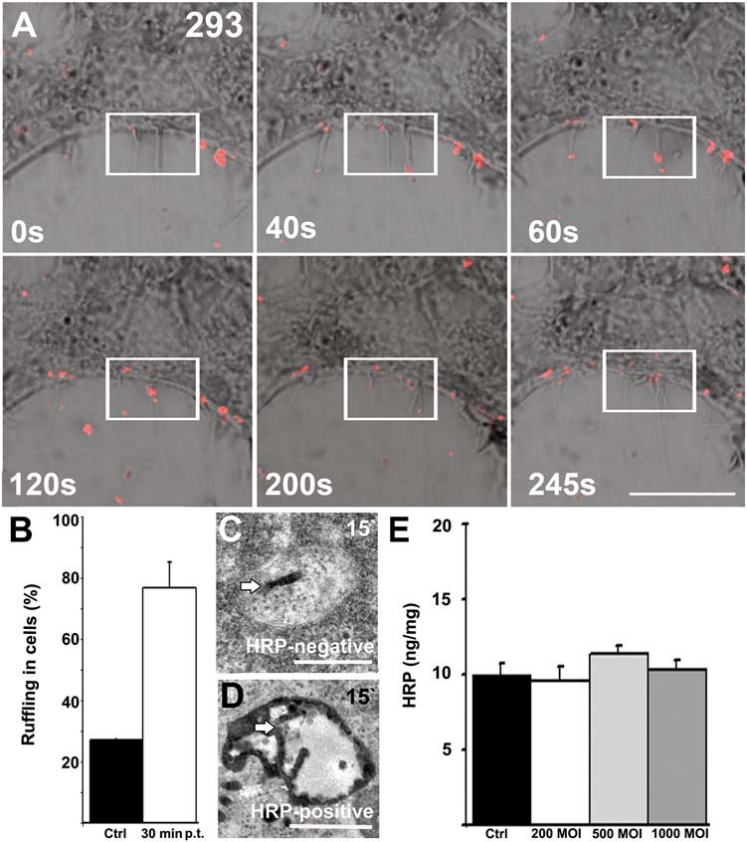
Baculovirus induces ruffling and internalizes along with fluid-phase markers. (A) Still images of baculovirus (p24mCherry, MOI 400) internalization in living 293 cells by confocal microscopy. Differential contrast image, baculovirus (red) and fixed time frames (0–245 s) are shown. Imaging was started at 5 min p.t. (0 s = 5 min). Some of the cellular protrusions guiding the baculovirus to the peripheral cytoplasm are within the rectangular box. Scale bar, 10 µm. (B) Cells positive for ruffles were calculated from control and baculovirus -treated cells 30 min p.t. (100 cells calculated). Mean values and standard deviations are shown. (C–D) Co-internalization of baculovirus (wt, MOI 500) with the fluid-phase marker HRP (10 mg/ml) was studied in 293 cells between 5 and 30 min p.t. HRP-negative (C) and positive (D) vesicles containing baculovirus (arrows) at 15 min p.t. are presented. Scale bar, 500 nm (C, D). (E) HRP (2 mg/ml) uptake after 30 min was measured with or without various amounts of baculovirus (200, 500 and 1000 MOI) co-internalized in cells. The amount of HRP is normalized to cellular protein content. Mean values and standard deviations are shown.

The binding and entry of the baculovirus (0–15 min p.t.) was next studied in live 293 and HepG2 cells by confocal microscopy. Interestingly, extensive ruffle formation on the cell surface of both cell types was detected early after administration of the virus (Figure 2A, Figure S2). The virions seemed to utilize the extended cellular protrusions for their attachment and further movement to the plasma membrane (Video S1). Additionally, engulfment of viruses from cellular ruffles was detected (Figure 2B). The cellular protrusions as well as cell surface areas, which were active in virus entry, were strongly positive for actin labeled with phalloidin rhodamine (Figure S2). Quantification of cells positive for ruffles showed that under control conditions 27% of the cells (±0.4% SD) showed ruffles, whereas at 30 min p.t. the number of cells showing ruffles was increased to 77% (±9% SD; n = 100).

Since many efficient uptake pathways have been connected recently with fluid-phase endocytosis, baculovirus was cointernalized with fluid-phase markers to human cells. Analyzed by EM, cellular ruffles and large endosomes were detected in baculovirus transduced 293 (Figure 2C) and HepG2 cells. The fluid-phase marker horseradish peroxidase (HRP) was detected in baculovirus-filled endosomes at 15 min p.t. in both cell types (Figure 2D) whereas at 5 min p.t., a majority of the endosomes were free of HRP and thus possibly connected with the plasma membrane. The ratios of HRP-positive vesicles containing baculovirus after 5, 15 and 30 min p.t. were 23.3%±11.7% (SD), 58.8%±9.1%, and 59.3%±10.1%, respectively. Measurement of the size of HRP- and baculovirus-filled endosomes by EM revealed that the structures were relatively large in diameter (603 nm±25 nm). Due to the high amount of ruffling and the large size of the endosomes, we next studied whether the fluid-phase uptake was inducible by baculovirus transduction, which is typical for macropinocytic uptake. HRP was allowed to internalize for 30 min alone or together with varied amounts of wild-type (wt) baculovirus (200, 500 and 1000 MOI; Figure 2E). After 30 min, cells were extensively washed with BSA containing buffer in order to remove the plasma membrane-bound HRP. The measurement of HRP activity after homogenization showed clearly that there was no difference in the uptake of HRP in cells with or without baculovirus transduction (Figure 2E). These results thus suggest that baculovirus induces ruffling and is internalized along with fluid-phase markers in large endosomes but does not itself induce fluid-phase uptake.

### Plasma membrane rafts are involved in baculovirus uptake

**Figure 3:**
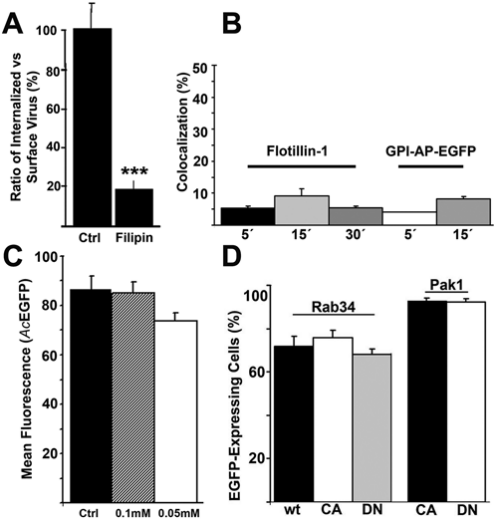
Plasma membrane rafts and macropinocytosis in baculovirus uptake. (A) The ratio of surface-bound (baculovirus Ab, A555) vs. intracellular baculovirus (baculovirus Ab, A488) was measured after 30 min p.t. during treatment with filipin in HepG2 cells from three separate samples (30 cells). Confocal sections were analyzed by BioimageXD. Mean values and standard deviations are shown. (B) Quantification of baculovirus colocalization with Flotillin-1 or GPI-AP-EGFP were analyzed by confocal microscopy in 293 cells after 5, 15 or 30 min p.t. from 30 cells from three separate experiments. Mean values and standard deviations are shown. (C) Macropinocytosis inhibitor EIPA (0.05 mM and 0.1 mM) was tested for its effects on baculovirus-mediated (*Ac*-EGFP, MOI 200) EGFP expression. Mean values of fluorescence intensity and standard deviations from FACS analysis are shown. (D) Baculovirus mediated EGFP expression was quantified at 6 h p.t. in the presence of transfected (48 h) macropinocytosis regulators Rab34 and Pak1 in 293 cells. The proportion of nuclei positive for EGFP expression of transfected cells was calculated from two separate experiments by confocal microscopy. In all studies, p-values were determined by unpaired Student's *t* test with a two-tailed *P* value. *P<0.05, **P<0.01, ***P<0.001.

Since several fluid-phase pathways are known to originate from the plasma membrane raft areas, we investigated whether internalization of the baculovirus is affected by drugs that interfere with the cholesterol content or affect its function on the plasma membrane. Since methyl-beta cyclodextrin has effects also on the clathrin pathway we used filipin, which has been shown to specifically affect raft and caveolae pathways [25]. The internalization assay showed that filipin inhibited the uptake of baculovirus by 82% (Figure 3A).

To elaborate on these results, we further tested the role of dynamin-independent, raft-derived GPI-anchored protein-enriched early endosomal compartment (GEEC; [26]) and flotillin pathways in baculovirus transduction. Flotillin-1 has been recently shown to define a dynamin and clathrin-independent entry pathway from the raft areas in mammalian cells [27]. In 293 or HepG2 cells, flotillin-1 and baculovirus showed no colocalization between 5 and 30 min p.t (Figure 3B, Figure S3A). Additionally, the colocalization of baculovirus with glycosyl-phosphatidylinositol (GPI)-anchored protein GPI-EGFP was investigated. GPI-EGFP was chased to the plasma membrane of 293 cells by a 4 h treatment with cycloheximide before viral administration. No apparent colocalization between baculovirus and GPI-EGFP was observed at 5–15 min p.t. (Figure S3B). Moreover, wt, CA or DN mutant forms of Cdc42, which is required for targeting GPI-anchored proteins further to early endosomes, showed no inhibiting effect on the cytoplasmic internalization of baculovirus in 293 cells (Figure S3C, S3D, S3E). Furthermore, immunolabeling of caveolin-1 and baculovirus in 293 cells after 5, 15 and 30 min internalization showed no detectable colocalization (Figure S1G). The results indicate that the baculovirus is internalized from the raft areas but does not use caveolae, GEEC or flotillin pathways for its uptake.

### Macropinocytosis is not involved in functional baculovirus entry

Since the baculovirus was observed in cell surface ruffles and in large cytoplasmic endosomes, the involvement of alternative uptake mechanisms including macropinocytosis was studied [12]. Here, different inhibitors and proteins involved in macropinocytosis were tested (Figure 3C and 3D). First, the effect of EIPA, an inhibitor of the Na^+^/H^+^ exchanger, which is frequently associated with macropinocytic uptake, was analyzed. No decrease in baculovirus-mediated marker gene expression was observed even at rather high concentrations of EIPA (0.05–0.1 mM; Figure 3C). The functionality of EIPA in these concentrations was verified by its inhibitory effect on entry of the fluid-phase marker TRITC-De (Figure S4).

Next, more specific regulators of macropinocytosis, namely Rab34 [28] and the p21-regulated kinase-1 (Pak1) [29] were studied. The DN and CA forms of Rab34 did not affect viral transduction efficiency at 6 h p.t. in 293 cells (Figure 3D, Figure S3K, S3L, S3M, S3N, S3O). As a control, the internalization of TRITC-De into Rab34 DN mutant transfected 293 cells was partially inhibited, whereas wt and CA constructs allowed more efficient internalization of De (wt 64.3%±6.0% SD, CA 60.2%±0.3% SD). Moreover, Pak1 had no effect on baculovirus transduction in 293 cells transfected with CA or DN mutants (Figure 3D). Control studies assured that the entry of TRITC-De into DN transfected 293 cells was partially inhibited (19.7%±0.20% SD), while CA transfected cells allowed more efficient internalization (39.8%±3.39% SD). In addition, we tested the effect of CtBP1/BARS (c-terminal binding protein 1/brefeldin A ADP-ribosylated substrate) for baculovirus entry. CtBP1/BARS was shown recently to regulate macropinocytosis donwstream of Pak1 kinase, which activates CtBP1/BARS by phosphorylation [30]. Thus we first verified that the DN CtBP1/BARS mutant construct was functional by preventing TRITC-De entry. The cells transfected with the WT CtBP1/BARS construct showed that 93% of the cells allowed dextran entry whereas the uptake was inhibited in 73% of the DN cells (inhibited cells showed less than 3 vesicles of dextran per cell). The results with baculovirus showed that the DN mutant construct (NBD-YFP) had no inhibitory effect on nuclear entry in 293 cells (Figure S3I, S3J). Similar results were gained in A431 cells (77.1% and 75.5% for NBD-YFP and BARS WT-YFP, respectively; over 100 cells calculated), which were used for CtBP1/BARS studies by Liberali et al (30). The data altogether suggest that the functional entry of baculovirus is not directly associated with macropinocytosis.

### Arf6-GTPase regulates baculovirus uptake and transduction

**Figure 4:**
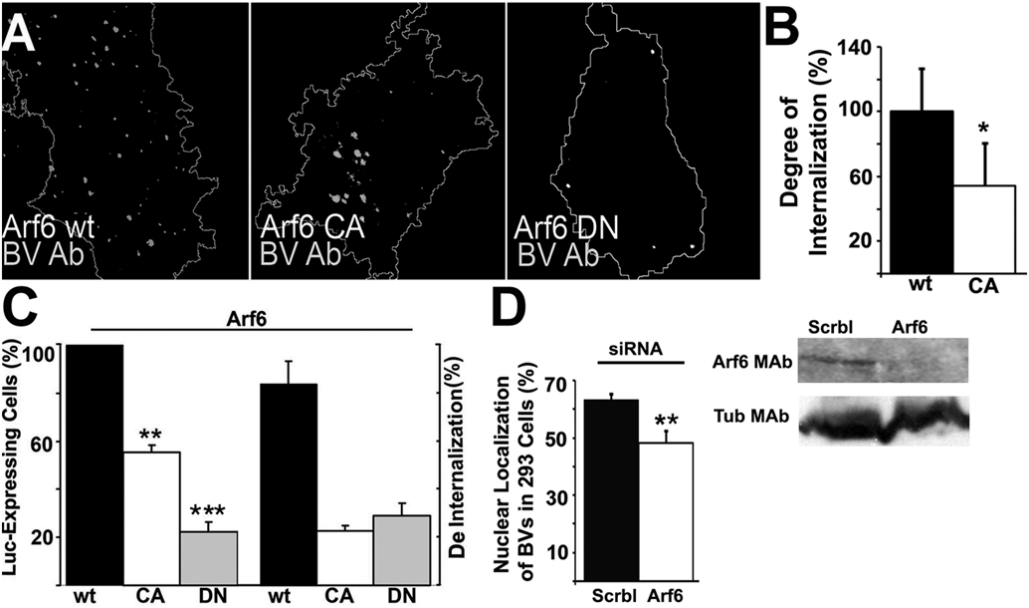
Arf6 is involved in baculovirus internalization and transgene expression. (A) 293 cells were transfected with Arf6 constructs (wt, CA, DN) for 48 h and then transduced with baculovirus (*Ac*VP39 MOI 200). The virus uptake after 30 min was detected by baculovirus Ab and Alexa-555-conjugated secondary antibody. The cell boundaries were determined from DIC image by ImageJ. The ratio of internalized vs. surface baculovirus in WT or Arf6DN transfected cells (30 cells from three separate experiments) was calculated from confocal sections using a differential labeling of baculovirus before and after permeabilization and using an internalization algorithm in BioimageXD software (see Materials and Methods section). Mean values and standard errors are shown. (B) Baculovirus-mediated luciferase (Luc; *Ac*-luc, MOI 200) expression in 293 cells was measured after 24 h in the presence Arf6 plasmids (left columns). Dextran internalization (2 h p.t.) in Arf6 expressing cells was measured to verify the efficacy of the mutant plasmids (right columns). The results show the proportional amount of cells which showed significant amount of dextran entry (cells with at least 10 dextran-positive vesicles in the cytoplasm). Mean values and standard deviations are calculated from three separate experiments. (C) Arf6 was knocked down in 293 cells using siRNAs. After siRNA treatments nuclear localization of baculovirus was calculated after 6 h baculovirus transduction. Western blotting was used to monitor the knock down effect with Arf6 antibodies. Statistical significance was determined by using the unpaired Student *t* test with a two-tailed *P* value. *P<0.05, **P<0.01, ***P<0.001.

The ADP-ribosylation factor 6 (Arf6) plays an important role in regulating clathrin-independent entry and recycling [31], [32]. In this study, 293 cells were transfected with the wt, CA or DN mutant forms of Arf6. In the presence of wt Arf6 protein, baculovirus (30 min p.t.) was efficiently internalized (Figure 4A). However, in cells transfected with the CA or DN forms of Arf6, the amount of internalized, cytoplasmic viruses was reduced. In order to reliably measure the internalization of baculovirus in the cells transfected with the DN construct, we performed an internalization assay in which the baculovirus was labeled with virus antibodies before and after permeabilization [33] (Figure 4A). This quantification showed that the internalization of baculovirus was reduced in DN transfected cells in comparison with wt Arf6 transfected cells (Figure 4B). Interestingly, in DN mutant cells, the percentage of baculovirus-mediated luciferase expression in the nucleus was decreased by 5-fold (Figure 4C) in comparison to cells transfected with the wt form of Arf6. Therefore, the results suggest that the DN Arf6 may have some additional inhibitory effect after internalization, on the translocation step to the nucleus. Furthermore, a 2-fold decrease was detected in nuclear entry in 293 cells transfected with the CA mutant (Figure 4C). In control studies, internalization of TRITC-De into DN and CA transfected cells was inhibited, while wt Arf6 allowed normal internalization verifying the importance of Arf6 regulating the fluid-phase entry (Figure 4C). We also used a siRNA approach to lower the expression of endogenous Arf6. Simultaneous transfection with a SiGlo transfection marker was used to pinpoint cells successfully transfected with siRNAs. In SiGlo positive cells, baculovirus internalization into 293 cell nuclei revealed a subtle decrease (Figure 4D) compared to the entry in scramble siRNA transfected and transduced cells at 6 h. Western blotting confirmed a relatively efficient knock-down (89%) of Arf6 expression in siRNA transfected cells. These results suggest that baculovirus uptake is regulated by Arf6, a regulator of clathrin-independent entry.

### RhoA regulates baculovirus uptake and transduction

**Figure 5:**
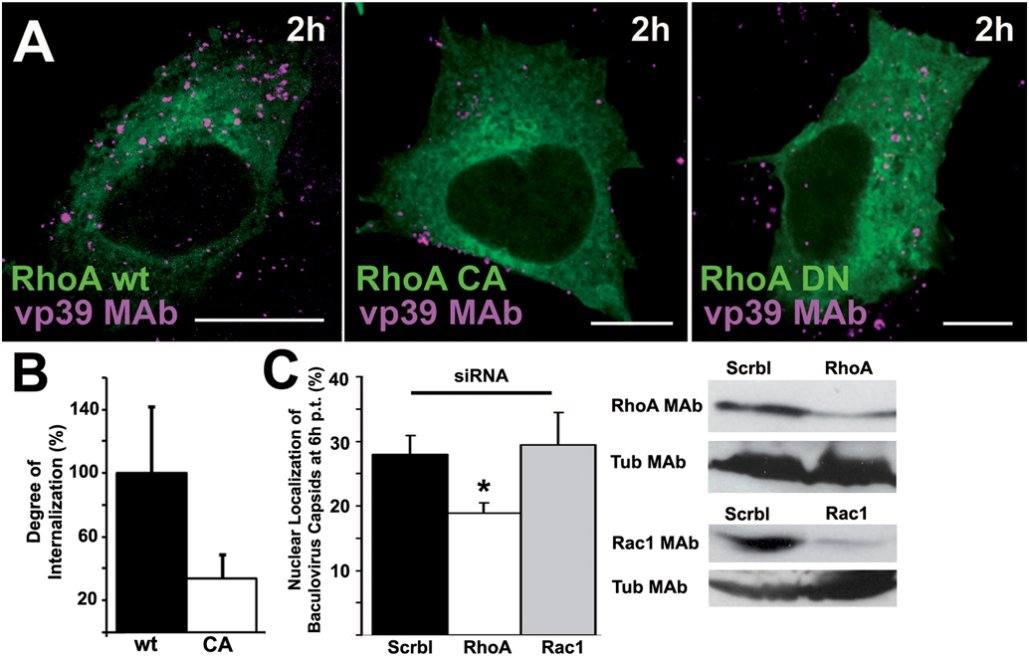
RhoA is involved in BV internalization and transgene expression. (A) BV internalization (wt, MOI 200) in 293 cells transfected for 24 h with wt, CA or DN RhoA-EGFP. The virus uptake (2 h) was detected by BV capsid antibody vp39 MAb (red) and A555-conjugated secondary antibody. Scale bars 10 µm. (B) Ratio of internalized vs. surface BV after RhoA wt and CA construct transfections was calculated from confocal sections after 30 min p.t. using a differential labeling of BV before and after cellular permeabilization (see Materials and Methods sections). (C) The effect of control (scrbl), RhoA or Rac1 siRNA treatments on BV nuclear entry was monitored in 293 cells (6 h p.t., wt, MOI 200). After immunolabeling, the proportion of SiGlo-positive nuclei positive for BV was measured from three separate samples (50–100 cells/each) by confocal microscopy. SiRNA knock down effects were monitored by western blotting with specific antibodies against RhoA (24 kDa) and Rac1 (26 kDa). In all quantitative images, mean values and standard errors are shown. Statistical significance was determined by using the unpaired Student's *t* test with a two-tailed *P* value, *P<0.05.

RhoGTPases modify actin filaments and are key regulators of cell growth, cell cycle progression and cell survival. Since actin is supposedly involved in the early events of baculovirus uptake causing e.g. ruffling, we tested the involvement of RhoGTPases in baculovirus entry. A preliminary inspection of 293 cells transfected with RhoA EGFP mutants showed that the cells transfected with the CA and DN mutants contained lower cytoplasmic amounts of baculovirus at 2 h p.t. (Figure 5A). Additional live imaging of RhoA CA mutant and fluorescent virus verified that the virus entry was decreased (1 h p.t., 2.5-fold reduction). The internalization of A546-TF and TRITC-De into cells transfected with the CA mutant was clearly inhibited (50.4%±12.6% SD and 79.8%±8.00% SD, respectively) in contrast to wt transfected cells, in which A546-TF and TRITC-De were efficiently internalized (91.8%±6.3% SD and 93.7%±2.8% SD, respectively). In order to verify further that the internalization of baculovirus was truly reduced in RhoA CA mutant cells, an internalization assay using differentially labeled baculoviruses before and after cell permeabilization was performed (Figure 5B). Quantification of viral internalization showed that in RhoA CA transfected cells the virus uptake was clearly decreased (ratio of internalized vs. surface baculovirus; Figure 5B).

To study further the role of RhoA in baculovirus entry, the endogenous expression of RhoA was reduced by siRNA (Figure 5C). In SiGlo positive cells, where the siRNA transfection was effective, viral internalization into 293 cell nuclei was clearly decreased at 6 h p.t. compared to the entry into cells treated with the scramble siRNA (Figure 5C). Western blotting confirmed a 70% knock-down of RhoA expression in siRNA transfected cells.

Since RhoA seemed to have an effect on baculovirus entry, we also tested the role of dynamin- and RhoA-dependent interleukin-2 (IL2)-receptor pathways [34]. After transfection with the Ntb-domain of IL2-receptor, the localization of internalized receptor was studied with respect to that of baculovirus. The confocal microscopy results showed that the surface-bound Ntb-detecting antibody did not colocalize with baculovirus in 293 cells between 5 and 60 min p.t. (Figure S3H), allowing us to conclude that baculovirus does not use the IL2 receptor pathway.

Next, we evaluated the influence of Rac1, another RhoGTPase family protein, on baculovirus transduction. In siRNA experiments against Rac1, nuclear entry of baculovirus at 6 h p.t. showed no statistically significant difference between Rac1 or scramble siRNA transfected cells (Figure 5C). Western blotting confirmed a 65% knock-down of Rac1 expression in siRNA transfected 293 cells. Due to the incomplete knock down, the negative results are merely suggestive, not conclusive. Further, at 2 h p.t. baculovirus showed normal internalization to both CA and DN Rac1-EGFP transfected 293 cells as detected by confocal microscopy (Figure S3F, S3G). In control studies, internalization of TRITC-De into transfected DN cells was inhibited, in contrast to CA transfected cells, where TRITC-De was efficiently internalized. The internalization of TF was efficient with both constructs (data not shown). Altogether, the results suggest that RhoA regulates baculovirus entry in contrast to Rac1.

### Phagocytosis-like uptake of baculovirus

**Figure 6:**
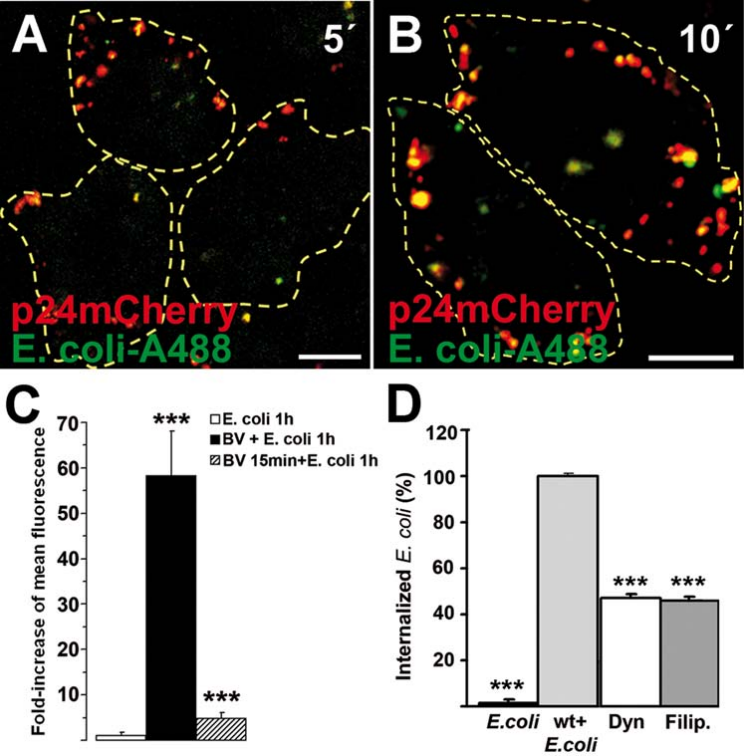
Phagocytosis-like uptake of baculovirus. (A,B) Baculovirus (p24mCherry, MOI 200; red) internalized together with A488-labeled *E. coli* bioparticles (green) in HepG2 cells at 5 (A) and 10 min (B) p.t. Scale bars, 10 µm. (C) Induction of phagocytic uptake of *E. coli* during baculovirus transduction. Fluorescent *E. coli* were fed simultaneously with baculovirus (MOI 200; baculovirus +*E. coli*) into cells and fixed at 60 min p.t. As a control, *E. coli* particles were fed into cells without baculovirus (*E. coli*) or after virus transduction for 15 min (baculovirus 15′+*E. coli*). To separate the fluorescence of internalized and non-internalized particles, the cells were treated with trypan blue. Normalized mean fluorescence values (Ctrl = 1) and standard deviation from 250–300 cells are shown. Fluorescence intensity was measured from confocal microscopy images (see Materials and Methods). (D) Dynasore and filipin were tested for their effects on stimulated *E. coli* uptake during baculovirus transduction. Co-internalization of baculovirus and E. Coli for 1 h without drugs was set to 100%. Fluorescence intensitites were calculated from three separate experiments (30–40 cells) using segmentation tools embedded in the BioimageXD software. Dyn (dynasore, 80 µM) and Filip. (filipin, 1 µg/ml) were added 30 min before the experiment and they were present during the whole internalization assay. Statistical significance was determined by using the unpaired Student's *t* test with a two-tailed *P* value, ***P<0.001.

Due to the large size of baculovirus, the induced ruffle formation, and the involvement of actin [34], Arf6, RhoA, as well as rafts in baculovirus transduction, the possible involvement of phagocytosis-like mechanisms in baculovirus entry was studied. For these experiments, we used heat-inactivated, Alexa-488-labelled *E. coli* (K12 strain, >1 µm) bioparticles, widely used as a marker of phagocytosis. First, we internalized *E. coli* particles and fluorescent baculovirus together for brief periods of time. The confocal results showed clear colocalization at 5 and 10 min p.t. in HepG2 cells (Figure 6A and 6B). Only a few *E. coli* particles were observed outside baculovirus-filled endosomes. We then monitored the intensity of fluorescent intracellular *E. coli* particles in baculovirus transduced cells by confocal microscopy. As a control, *E. coli* alone was fed to HepG2 cells for 1 h (Figure 6C). To separate the fluorescence of internalized and non-internalized particles, the cells were treated with trypan blue in order to quench the extracellular fluorescence. Untransduced control cells contained no apparent fluorescence after 1 h treatment with *E. coli* particles suggesting that bacteria did not enter the cells without baculovirus. Similarly, in 293 cells *E.coli* alone did not enter the cells and gave only low background fluorescence (data not shown). In contrast, virus-transduced HepG2 and 293 cells contained high amounts (49-fold and 10-fold more, respectively) of internalized *E. coli* particles (*P*<0.001), indicating that virus could induce entry of bacteria in non-phagocytic human cells. Interestingly, when baculovirus was first fed to HepG2 cells for 15 min and then *E. coli* for the next 60 min, bacteria could no longer enter cells efficiently. These results suggest that baculovirus is able to induce transient bacterial entry when administered simultaneously.

We also performed an *E. coli* internalization assay with dynasore and filipin in order to test whether the baculovirus stimulated uptake of *E. coli* occurs in a similar manner in drug-treated cells (Figure 6D). The assay showed that both filipin and dynasore caused a significant decrease of the stimulated uptake, suggesting that the entry of *E. coli* is also dynamin-dependent and originates from the raft membranes.

## Discussion

We demonstrated previously that baculovirus accumulates into EEA1-positive endosomes in HepG2 cells starting at 30 min p.t. [12], [13]. High frequency of ruffles and the presence of large smooth-surfaced endosomes full of baculovirus indicated the involvement of a more efficient entry pathway than the previously suggested clathrin-dependent uptake [12], [15]. Since clathrin-coated vesicles (100–150 nm) and caveolae (50-80 nm) are thought to have a rather rigid coat structure and invariable, uniform size in different cell lines [35], the size of the virus itself (approx. 30-60 nm diameter×250–300 nm length [36]), may limit its internalization into human cells. However, recent results revealed, strikingly, that even large bacteria are able to recruit clathrin and utilize the pathway for their entry [37], [38]. Therefore, clathrin structures may serve as one real alternative route for large virus particles. In this study, careful colocalization measurement by confocal microscopy showed no association of baculovirus with the clathrin heavy chain during viral transduction. Moreover, baculovirus does not associate with transferrin or recycling endosomal marker Rab11 [12]. Additionally, baculovirus containing clathrin-coated vesicles were not observed in our EM studies. Our results also showed that the early entry is sensitive to the cholesterol aggregating drug filipin suggesting that the raft domains are important for the first uptake step. Several viruses using the CME have been shown to be unaffected by the filipin-treatment, including Sindbis virus, West Nile virus and papillomavirus [39], [40], [41].

Recently, Long et al. [15] suggested a role for CME in the baculovirus transgene expression in BHK21 cells based on the effects of chlorpromazine and on the DN mutant form of Eps15. However, the initial entry step was not studied in detail [15]. In this study, we observed that the expression of DN Eps15 did not inhibit the actual viral entry step in 293 cells, indicating an inhibiting step later in the pathway leading to decreased transgene expression (Figure S1C, S1D). In addition, chlorpromazine did not have a significant effect on baculovirus entry in our assays. If the formation of clathrin coated pit (that is inhibited by chlorpromazine) would be crucial for baculovirus entry, it should have been also obvious in our clathrin colocalization studies as well as in our EM observations. Our results altogether suggest that baculovirus does not use the clathrin-mediated entry to efficiently express its transgenes in the human cells used in this study, but rather a clathrin-independent entry mechanism. We cannot, however, exclude the possible differences between the cell types.

As more data accumulates on ligands using clathrin- and caveolin independent carriers [23], [42] there is increasing evidence that these pathways show differential usage of cellular regulators such as dynamin and RhoGTPases. Clathrin- and caveolin independent pathways include e.g. the dynamin-dependent IL-2 receptor pathway and the dynamin-independent GEEC and flotillin pathways. The GEEC pathway carrying GPI-anchored proteins is dependent on Cdc42 for early endosomal targeting [26]. Dynamin-independent flotillin, on the other hand, defines its own pathway and does not seem to share any similarities with the GEEC pathway [27]. Our data on RhoGTPases in 293 cells suggest that baculovirus entry is independent of Rac1 and Cdc42. Moreover, only RhoA had an effect on baculovirus uptake. RhoA has been linked with clathrin- and caveolin-independent but dynamin-dependent entry, such as is the IL2-receptor pathway [34]. However, the observed lack of colocalization with the IL2-receptor implies that the entry of baculovirus does not involve the IL2-receptor pathway. Moreover, baculovirus did not colocalize with internalized flotillin or with GPI-EGFP, suggesting that functional baculovirus entry is independent of GEEC and flotillin pathways as well. Interestingly though, overexpression of GPI-EGFP, Rac1 and Cdc42 did inhibit uptake of baculovirus to some extent (Figure S5). It may be possible that their expression boosts “competing entry pathways” e.g. the GEEC pathway, which may down-regulate other pathways, such as that used by baculovirus. This effect has previously been observed between raft-derived and clathrin-dependent pathways when siRNAs against effectors of clathrin route boosted raft-derived pathway and vice versa [43].

Macropinocytosis is a form of clathrin- and caveolin-independent uptake, which has very little in common with other caveolin- and clathrin-independent pathways but shows many similarities with phagocytosis [44]. Macropinosomes may form spontaneously or they may be elicited by growth factors and phorbol esters. Macropinocytosis relies on molecules such as RhoGTPase Rac1, p21-kinase Pak1, CtBP1/BARS, PI3K, PLC, GTPase Rab34 and actin for entry [30], [45]. Macropinocytosis has also been associated with frequent actin-driven ruffles on the plasma membrane. A few bacteria and viruses, such as *Shigella flexneri*, *Salmonella typhimurium*, *Haemophilus influenzae*, HIV-1 and adenovirus 3 have been shown to rely on macropinocytosis for their entry [46], [47], [48], [49]. In contrast to our expectations, the transduction of baculovirus was not regulated by Rac1, Pak1, CtBP1/BARS, Rab34 proteins or EIPA. Furthermore, baculovirus treatment did not induce fluid-phase uptake which, altogether, suggest that baculovirus is not internalized by a macropinocytic process.

Recent data from bacterial pathogens suggests that various particles may be ingested together with fluid by a macropinosome-like phagocytic process [44]. Ruffles form a loose fitting phagosome, which close up to form a phagocytic cup. Regulators of movement and actin polymerization show several shared features among macropinocytosis and phagocytosis and they include members of RhoGTPases, such as Rac1, Cdc42, Arf6 and RhoA. It was recently shown that CtBP1/BARS phosphorylated by Pak1 is used for the closure of the macropinocytic cup [30]. In the case of baculovirus, we found no connection to CtBP1/BARS or Pak1, which further supported our conclusion that baculovirus entry does not follow macropinocytosis. In contrast, for functional baculovirus transduction, two regulators were identified, Arf6 and RhoA. Previously, Arf6 GTPase was shown to facilitate the phagocytic uptake of red blood cells in macrophages and entry of a small bacterium Chlamydia by reorganizing actin [50]. In our study, baculovirus uptake and transgene expression were affected by DN and CA mutants or specific siRNA of Arf6. Inhibition of entry by the CA mutant may have, however, occurred through inactivation of RhoA. This effect has previously been shown by Boshans et al. [51], who demonstrated that activation of Arf6 downregulates RhoA signaling and depletes stress fibers in CHO cells.

A recent study on mimivirus showed that professional phagocytes may engulf large viruses using a phagocytic mechanism [52]. In addition to professional phagocytes, such as macrophages, dendritic cells and polymorphonuclear leucocytes, many other cell types are able to engulf material by a phagocytic mechanism [53]. In a recent interesting study, Herpes Simplex virus 1 was demonstrated to use phagocytosis-like uptake regulated by RhoA but not Cdc42 or Rac1 in professional and non-professional phagocytes [54]. Although phagocytosis-mediated engulfment is expected to be induced only by particles larger than 0.5 µm [55], [56], the study showed that Herpes Simplex virus (0.17–0.2 µm), close to the diameter of baculovirus, activated the entry of phagocytic tracer *E. coli* bioparticles [54]. Previous work has linked RhoA, but not Cdc42 or Rac1, with complement-activated phagocytosis [57]. Here, we show that baculovirus entry enhances ruffle formation on the cell surface and the uptake of *E. coli* bioparticles in non-phagocytotic epithelial mammalian cells. Phagocytosis is suggested to be a dynamin-dependent and raft-derived process on the plasma membrane [58]. Similarly, baculovirus early uptake was sensitive to dynasore and to drugs that affected raft domains. Filipin also showed similar inhibition in stimulated *E. coli* uptake. The stimulated *E. coli* uptake followed similar regulation as baculovirus uptake.

To conclude, in human cells, the cellular binding of baculovirus induces ruffle formation and engulfment of several baculovirus in large cellular invaginations mainly in the raft areas. The functional entry of baculovirus occurs via clathrin-independent smooth-surfaced vesicles and does not involve raft-derived IL2-receptor, flotillin or GEEC pathways. The entry mechanism is reminiscent of phagocytosis, as it is regulated by dynamin, RhoA and Arf6 and as it induces the uptake of *E. coli* in non-phagocytic human cells.

## Materials and Methods

### Cells

Human hepatocarcinoma (HepG2) and human embryonic kidney (293) cell lines were obtained from the American Type Culture Collection (ATCC, Manassas, VA). The cells were grown in monolayer in Minimum Essential Medium (MEM) supplemented with 10% inactivated fetal calf serum (FCS), L-glutamine and penicillin-streptomycin (Gibco BRL, Paisley, UK) at 37°C, in 5% CO_2_. For HepG2 cells non-essential amino acids (Gibco BRL) and Na-pyruvate (Merck & Co. Inc., Whitehouse Station, NJ) were also used. *Spodoptera frugiperda* (*Sf*9; GibcoBRL, Grand Island, NY/CRL 1711, ATCC) insect cells were maintained in monolayer and suspension cultures at 28°C using serum-free Insect-XPRESS culture medium (Cambrex, Walkersville, MD) or HyQ®SFX-Insect medium (HyClone Inc, Logan, UT) without antibiotics.

### Viruses

Wild type (wt), *Autographa californica* nucleopolyhedrovirus (*Ac*MNPV; E2 strain), recombinant baculovirus vp39EGFP (Kukkonen et al. 2003), *Ac*-luc [59], CAG-BV-EGFP [60], *Ac*-EGFP and *Ac*VP39 [61] and p24mCherry were used. Shortly, p24mCherry virus was prepared by cloning baculovirus capsid protein p24-RFP (mCherry; [62] into pBACcap-1 vector [13]. The concentrated batches of viruses were prepared and the virus titers were gained as described previously [63]. As the virus dose, MOI 200 was used in all mammalian cell transductions unless otherwise stated.

### Antibodies and chemicals

Monoclonal antibodies (MAb) against the following proteins were used: Photinus pyralis luciferase MAb (Serotec, Oxford, UK), early endosome antigen 1 MAb (eea-1; Transduction Laboratories, Lexington, KY, UK), myc MAb (9E10, ATCC), flotillin-1 MAb (BD Biosciences, San Jose, CA), nuclear lamin A/C (Novocastra laboratories, Newcastle upon Tyne, UK), Arf6 MAb (Thermo Fisher Scientific, Fremont, CA), RhoA MAb (SantaCruz Biotechnology Inc., Santa Cruz, CA), Rac-1 MAb (Millipore, Billerica, MA), FLAG M2 MAb, tubulin MA (Sigma Aldrich, St Louis, MO) and *Ac*MNPV vp39 capsid protein MAb (Dr. L. Volkman, University of California, Berkeley, CA). Polyclonal antibodies (Ab) against the following proteins were used: Dynamin-2 Ab (Dr. M. McNiven, Mayo Clinic College of Medicine, Rochester, MN), NTb (IL-2):Cy3-561 (Dr. A. Dautry-Varsat, Pasteur Institut, Paris, France), actin Ab (Sigma Aldrich), clathrin heavy chain (Abcam) and *Ac*MNPV Ab (baculovirus Ab; Drs. S. Braunagel and M. Summers, Texas A&M University, TX). HRP (type II), filipin, amiloride (EIPA), chlorpromazine, TRITC-phalloidin and cycloheximide were from Sigma. TRITC-labeled dextran (TRITC-De, 10 kDa), Alexa-546- and Alexa-488-labeled transferrin (A546-TF, A488-TF) and A488-labeled *E. coli* (K-12 strain) bioparticles were from Molecular Probes (Eugene, OR). In the double-labeling studies, A488, A555 and A633-conjugated anti-mouse and anti-rabbit antibodies (Molecular Probes) were used. Dynasore (C_18_H_14_N_2_O_4_); synthesized by Dr. Henry E. Pelish, (Kirchhausen Lab, Immune Disease Institute, Boston, MA), was used at final concentration of 80 µM in serum free medium.

### Virus transduction

The cells were grown to subconfluency and transduced in MEM containing 1% FCS for 1 h at 4°C or 37°C followed by incubation in MEM containing 10% FCS at 37°C. For co-internalization studies, the cells were first transduced with virus (wt/vp39EGFP MOI 200–1000) for 15 min, washed, and then fed with TRITC-De (250 µg/ml, in 1% culture medium) for 5–45 min. A546-TF (200–250 µg/ml), FITC-De (1000 µg/ml) or A488-labeled *E. coli* bioparticles (60 particles/cell) were fed simultaneously with virus. In experiments with *E. coli*, virus (wt MOI 200) was also fed to cells for 15 min, followed by Alexa-488-labeled *E. coli* particles for 60 min. All samples were treated with trypan blue in order to separate the fluorescence of internalized and non-internalized *E. coli* particles. Additionally, in experiments with the GPI-EGFP construct, the cells were treated continuously with cycloheximide (100 µg/ml, diluted in 1% medium) for 4 h prior to virus transduction, in order to chase the expressed GPI-EGFP to the plasma membrane. To get rid of excess plasma membrane stain, the cells were further washed with 0.5 M NaCl, 0.2 M sodium acetate buffer (pH 4.5) and finally immunolabeled and detected with confocal microscopy. In inhibition experiments detected by confocal microscopy, the cells were preincubated with filipin (1 µg/ml) for 30–60 min, followed by baculovirus binding on ice for 30–60 min and transduction with or without drugs for 6–24 h at 37°C. The drug treatments did not affect cell viability as determined by CellTiter 96® Aqueous One Solution Cell Proliferation Assay (MTT assay; Promega) according to the manufacturer's protocol.

### Electron microscopy

For HRP labeling experiments, the cells were first incubated for 1 h on ice and/or 5–30 min at 37°C in complete culture medium containing 10 mg/ml HRP (Sigma, type II). After viral transduction (wt, MOI 500), the cells were fixed in 4% paraformaldehyde (PFA) containing 0.1% glutaraldehyde (in 50 mM Tris buffer, pH 7.6) for 1 h at RT. HRP was detected with 0.1% diaminobenzidine (Sigma) for 30 min, followed by another 30 min in 0.1% diaminobenzidine supplemented with 0.1% hydrogen peroxide. The cells were then washed, post-fixed and processed for EM.

### HRP uptake assay

HRP (2 mg/ml in DMEM containing 1% serum) was administered with or without various amounts (200, 500 and 1000 MOI) of wild type baculovirus on HepG2 cells. After a 30 min-internalization period cells were put on ice and extensively washed with PBS supplemented with BSA (0.5%) to remove of plasma membrane-bound HRP. EM samples with HRP labeling showed that such washes were sufficient. Cells were scraped from the dishes, centrifuged to give a cell pellet and treated with 1% Triton X-100 in PBS for 30 min on ice. Cell debris were centrifuged (5 min at 15 000 g) and discarded, whereas the supernatant was evaluated for its content of HRP activity [64] and protein (BIO-RAD Protein assay).

### Transfection experiments

293 cells were transfected for one to two days according to manufacturer's protocol (Fugene, Roche, Basel, Switzerland). In the experiments, the following constructs were used: myc-tagged Pak1T423E (Dr. J. Chernoff, Fox Chase Cancer Center, Philadelphia, PA), flag-tagged Pak1AID (Dr. E. Manser, GSK-IMCB Laboratory, Singapore), Cdc42 WT and GPI-EGFP (Dr. L. Pelkmans (ETH Zürich, Switzerland), Cdc42 and Rac1 (17N, 12V), Arf6-EGFP WT, Arf6-T27N, Arf6-Q67L, RhoA (WT, 14V, 19N; Dr. J. Peränen, University of Helsinki, Finland), Rab34 (WT, CA and DN; Dr. W. Hong, GSK-IMCB Laboratory, Singapore), clathrin-chain-tomato (Dr. T. Kirchhausen, Harvard Medical School, Boston, MA) and IL-2R beta-chain (NTb; Dr. A. Dautry-Varsat, Pasteur Institut, Paris, France). The effects of expression of the WT constructs of different proteins on virus uptake were compared to each other, as well as to virus upatake after mock transfection, and without any transfection (Figure S5). No major changes were revealed in the virus uptake due to most plasmid transfections. Interestingly though, overexpression of GPI-GFP, Rac1 and Cdc42 did inhibit uptake of baculovirus to some extent possibly by boosting competing entry pathways.

### Immunofluorescence labeling, confocal microscopy, and data analysis

Cells were fixed with 4% PFA for 20 min, permeabilized with 0.2% Triton X-100, immunolabeled according to standard protocols, and subjected to confocal microscopy (Zeiss LSM 510, Carl Zeiss AG, Jena, Germany or Olympus Fluo-View 1000, Olympus Optical Co., Tokyo, Japan). In the imaging, appropriate excitation and emission settings were used (488-nm argon laser, 543-nm and 633-nm HeNe-lasers). In live cell microscopy (Zeiss LSM 510) the objective and sample holder were heated to 37°C. Serial sections were obtained by using 60× APO oil immersion objective (NA = 1.35) or 63× Plan-Neofluor oil immersion objective (NA = 1.25) with a resolution of 512×512 pixels/image.

Quantification of internalization and colocalization was determined with a free, open source software package, BioImageXD [24] as described before [33]. Briefly, to quantify the level of colocalization, 30 cells from three independent experiments, 10 cells from each experiment, were randomly selected and optically sectioned using a confocal microscope. Colocalization was evaluated from the center slice of the cell by examination of the merged images, and analysis was performed with BioImageXD.

BioImageXD contains a simple algorithm for calculating the ratio of internalized/surface virus. The formula is: Ch1/(Ch2 – Coloc), where Ch1 = number of voxels stained after permeabilization, Ch2 = number of voxels stained before permeabilization, Coloc = number of colocalized voxels. Only voxels with intensity values above thresholds were considered for each of the three values. The thresholds were determined as above.

The amount of internalized *E. Coli* in cells was determined by intensity threshold segmentation. The threshold was selected so that only clear intracellular structures were visible. A connected component labeling algorithm was used to eliminate structures less than three voxels.

### Flow cytometry

The cells were first preincubated with or without drug-containing medium (EIPA 0.025–0.1 mM) for 30–60 min, followed by baculovirus binding on ice for 30–60 min and transduction with or without drugs for 24 h at 37°C. The possible interfering effect of cellular inhibitors on viral binding, or on the fluorescence signal were controlled. The samples were scraped or detached by trypsin-treatment and analyzed with FACSCalibur and CellQuest software (Beckton Dickinson, Heidelberg, Germany).

### RNAi

The following siRNA sequences (20 µM; Dharmacon, Thermo Fisher Scientific, Fremont, CA) were used: RhoA (5′-GAAGUCAAGCAUUUCUGUC-3′), Rac1 (5′-GAUAACUCACCACUGUCCA-3′), Arf6 (5′-GCACCGCAUUAUCAAUGACCGUU-3′) and dynamin2 (SMARTpool). Non-specific siRNA was used as a negative control, wheras siGLO (Dharmacon) acted as a transfection marker. Cells were transfected using Oligofectamine (Invitrogen) according to the manufacturer's instructions. Mock-transfected cells were treated with Oligofectamine alone. At 72 h transfection, the cells were transduced with baculovirus for 6 h, fixed and immunolabeled with virus capsid labeling antibody. After immunolabeling, virus capsid localization in SiGlo-positive nuclei was analyzed visually from three separate samples (50–100 cells/each) by confocal microscopy. The functionality of siRNAs was shown by SDS-PAGE and western blot analysis of the respective proteins. Tubulin was used as a loading control. Using ImageJ software, differential gel band intensities of scramble and target siRNAs were detected.

### Statistical testing

T-test was used for pairwise statistical comparison between samples. For percentages or ratio figures, t-test was applied after arcsin √ or logarithmic transformation of the original variable to convert the binomial distribution of the data to a normal distribution.

## Supporting Information

Figure S1 Clathrin-mediated endocytosis is not involved in baculovirus early uptake. (A,B) Effect of baculovirus transduction on clathrin localization pattern in 293 cells. By live confocal microscopy, the expressed, fluorescent light chain clathrin (clathrin light chain tomato) showed similar distribution after baculovirus transduction (C; wt, 1000 MOI, 60 min p.t.) as untransduced, transfected control cells (B). Pseudocolor images are presented for better visualization of the expressed clathrin. (C,D) The expression of wt (C) or DN (D) clathrin coat assembly regulator protein Eps15-EGFP (green) and internalization of baculovirus capsid (wt, vp39 MAb, 200 MOI; red) after 2 h p.t. in 293 cells. (E,F) Baculovirus (p24mCherry, 200 MOI; red) was internalized in untreated (E) or dynasore-treated (F) 293 cells for 30 min together with CME marker transferrin (A546-TF; red). (G) Baculovirus (p24mCherry, 200 MOI, red) localization in 293 cells with caveolin-1 MAb (green). Alexa-555 and -488 were used as secondary antibodies. In all images, scale bars 10 µm.
(4.90 MB TIF)

Figure S2 Baculovirus induces ruffle formation. (A) Cellular ruffle formation in live baculovirus transduced HepG2 cells. Baculovirus (MOI 400) was directly fed onto cells in the confocal microscope, and details of the cell surface protrusions were followed immediately during 0–15 min p.t. DIC images reveal protrusions growing from the cell surface. Baculovirus is not visualized for clarity. (B) Baculovirus was internalized for 15 min into 293 cells and, after PFA fixation, baculovirus capsid and actin were labeled by antibodies (Ab, Alexa-488, green) and TRITC-phalloidin (red), respectively. Scale bars, 10 µm.
(1.30 MB TIF)

Figure S3 Localication and entry of baculovirus after expression of various endocytic membrane traffic regulators. (A,B) Baculovirus (wt, MOI 200, red) localization with Flotillin-1 MAb at 15 min p.t. (A) or GPI-EGFP at 30 min p.t. (B) in 293 cells (green). Baculovirus (wt, MOI 200) entry into 293 cells transfected with the wt, CA and DN mutants of Cdc42-EGFP at 6 h p.t. (C–E), as well as DN and CA mutants of Rac1-EGFP at 2 h p.t. (F,G) (green). (H) 293 cells were transfected with the Ntb-domain of IL2-receptor visualizing the localization of internalized IL2-receptor after baculovirus (wt, MOI 200, red) transduction for 30 min. Ntb-detecting antibody conjugate (NtB(IL-2):Cy3-561; green) was added on the plasma membrane before baculovirus addition. (I–O) Baculovirus (p24mCherry, MOI 200) entry after 6 h p.t. in 293 cells transfected with the wt and DN mutant of CtBP1/BARS (BARS; I–J), CA and DN mutants of Pak1 (K,L) as well as wt, DN and CA mutants of Rab34 (M–O; green). In images A,B and F–H, baculovirus was labeled with capsid labeling antibodies vp39 MAb or BV Ab together with Alexa-555 secondary antibody (red). In images C-E and I-O, p24mCherry construct was used. In all images, scale bars 10 µm.
(9.89 MB TIF)

Figure S4 The effect of EIPA on fluid-phase entry. The efficacy of EIPA was tested by internalizing TRITC-De for 30 min in untreated (A) or EIPA-treated (0.1 mM) 293 cells (B). A representative group of cells are shown with (right) or without (left) DIC image merged with TRITC-De. Scale bars, 10 µm.
(2.63 MB TIF)

Figure S5 Putative effects on baculovirus entry due to expression of various endocytosis regulating WT constructs. Various plasmid constructs were transfected into 293 cells for 48 hours prior to baculovirus (p24mCherry, 200 MOI) uptake for 30 min. The plasma membrane-bound virus was extensively washed before the PFA fixation of the. Transfected cells (n = 300–400 cells) were scanned by confocal microscopy from three separate samples and analyzed for their p24mCherry fluorescence intensity. The results are shown as mean values ± SE. Untransfected cells (control) and cells expressing plain EGFP (GFP) were as controls.
(1.64 MB TIF)

Video S1 Baculovirus internalization into living 293 cells was observed by confocal microscopy. In the imaging, fluorescent (p24mCherry, MOI 400) virus was fed onto cells and the attachment and internalization of the viruses were monitored thereafter. Differential contrast image (DIC), virus (red) and selected time frames (0–400 s) are shown.
(3.92 MB MOV)
