# Supplementary figures and images for: Clathrin-Independent Entry of Baculovirus Triggers Uptake of E. coli in Non-Phagocytic Human Cells
Source: PLoS One. 2009 Apr 7;4(4):e5093. doi: 10.1371/journal.pone.0005093 (PMC2662421; doi:10.1371/journal.pone.0005093)

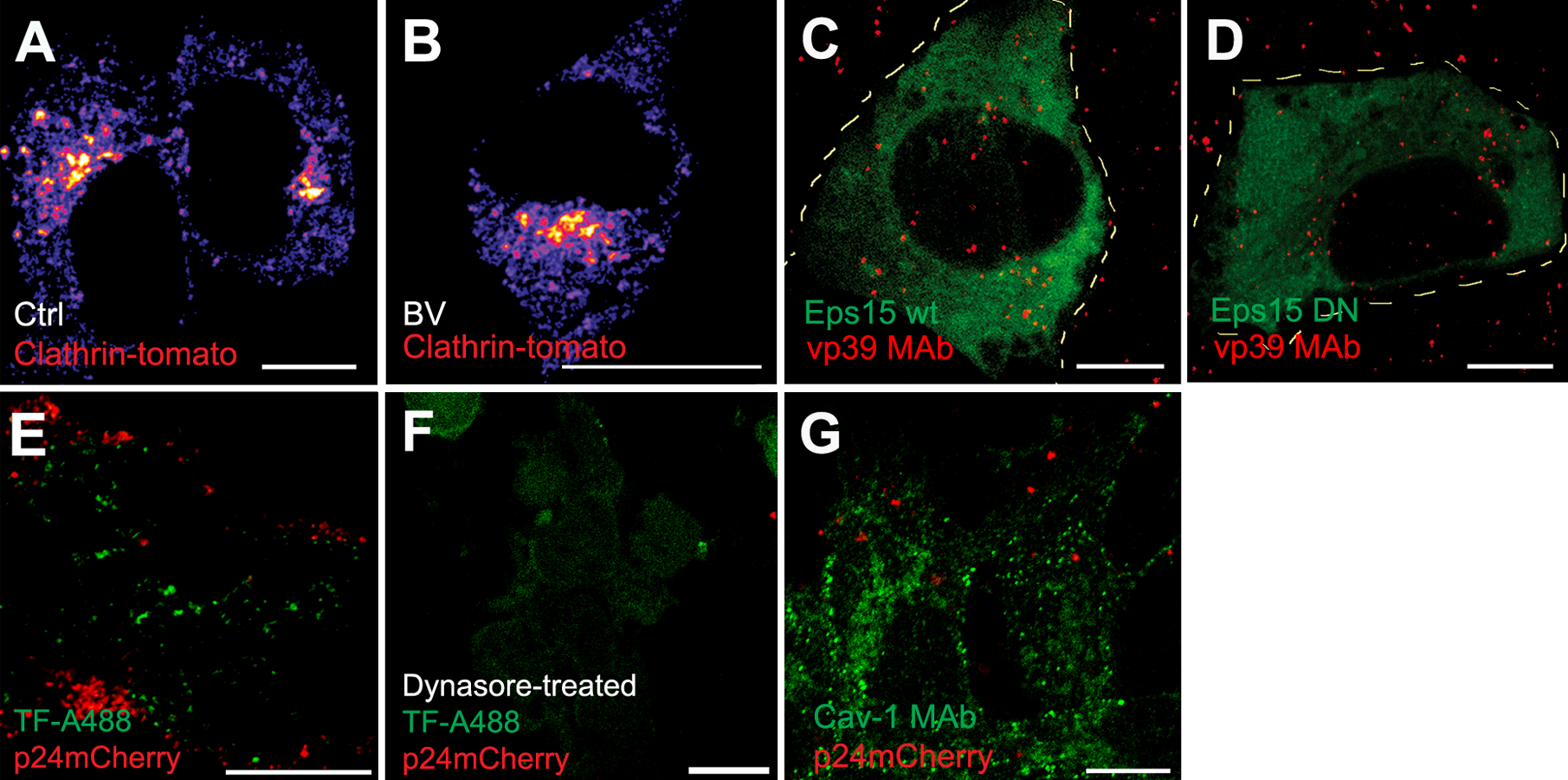

Supplement: Figure S1 — Clathrin-mediated endocytosis is not involved in baculovirus early uptake. (A,B) Effect of baculovirus transduction on clathrin localization pattern in 293 cells. By live confocal microscopy, the expressed, fluorescent light chain clathrin (clathrin light chain tomato) showed similar distribution after baculovirus transduction (C; wt, 1000 MOI, 60 min p.t.) as untransduced, transfected control cells (B). Pseudocolor images are presented for better visualization of the expressed clathrin. (C,D) The expression of wt (C) or DN (D) clathrin coat assembly regulator protein Eps15-EGFP (green) and internalization of baculovirus capsid (wt, vp39 MAb, 200 MOI; red) after 2 h p.t. in 293 cells. (E,F) Baculovirus (p24mCherry, 200 MOI; red) was internalized in untreated (E) or dynasore-treated (F) 293 cells for 30 min together with CME marker transferrin (A546-TF; red). (G) Baculovirus (p24mCherry, 200 MOI, red) localization in 293 cells with caveolin-1 MAb (green). Alexa-555 and -488 were used as secondary antibodies. In all images, scale bars 10 µm. (4.90 MB TIF) [file pone.0005093.s001.tif]

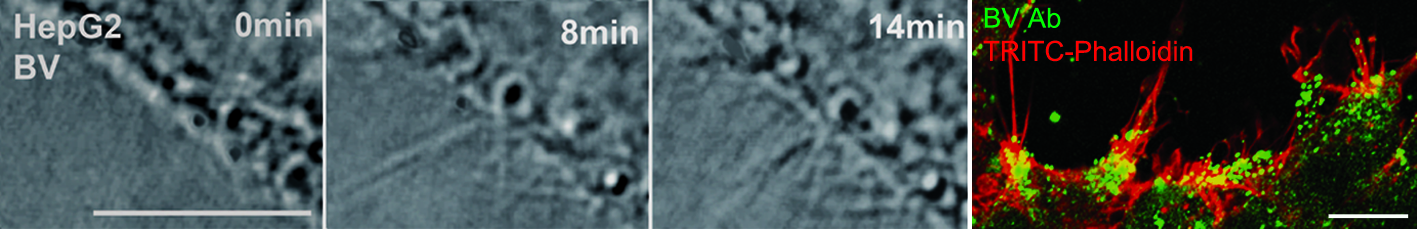

Supplement: Figure S2 — Baculovirus induces ruffle formation. (A) Cellular ruffle formation in live baculovirus transduced HepG2 cells. Baculovirus (MOI 400) was directly fed onto cells in the confocal microscope, and details of the cell surface protrusions were followed immediately during 0–15 min p.t. DIC images reveal protrusions growing from the cell surface. Baculovirus is not visualized for clarity. (B) Baculovirus was internalized for 15 min into 293 cells and, after PFA fixation, baculovirus capsid and actin were labeled by antibodies (Ab, Alexa-488, green) and TRITC-phalloidin (red), respectively. Scale bars, 10 µm. (1.30 MB TIF) [file pone.0005093.s002.tif]

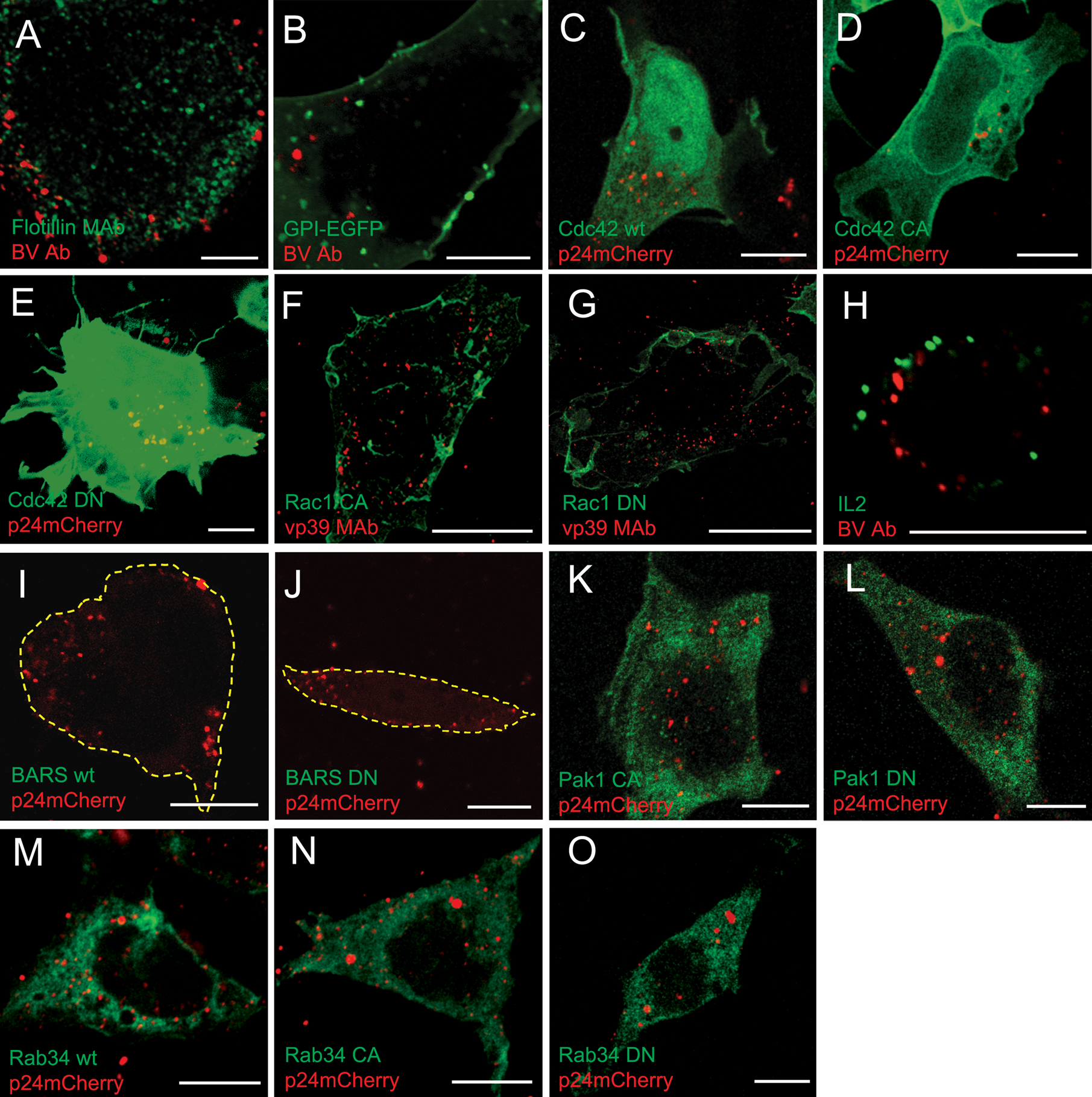

Supplement: Figure S3 — Localication and entry of baculovirus after expression of various endocytic membrane traffic regulators. (A,B) Baculovirus (wt, MOI 200, red) localization with Flotillin-1 MAb at 15 min p.t. (A) or GPI-EGFP at 30 min p.t. (B) in 293 cells (green). Baculovirus (wt, MOI 200) entry into 293 cells transfected with the wt, CA and DN mutants of Cdc42-EGFP at 6 h p.t. (C–E), as well as DN and CA mutants of Rac1-EGFP at 2 h p.t. (F,G) (green). (H) 293 cells were transfected with the Ntb-domain of IL2-receptor visualizing the localization of internalized IL2-receptor after baculovirus (wt, MOI 200, red) transduction for 30 min. Ntb-detecting antibody conjugate (NtB(IL-2):Cy3-561; green) was added on the plasma membrane before baculovirus addition. (I–O) Baculovirus (p24mCherry, MOI 200) entry after 6 h p.t. in 293 cells transfected with the wt and DN mutant of CtBP1/BARS (BARS; I–J), CA and DN mutants of Pak1 (K,L) as well as wt, DN and CA mutants of Rab34 (M–O; green). In images A,B and F–H, baculovirus was labeled with capsid labeling antibodies vp39 MAb or BV Ab together with Alexa-555 secondary antibody (red). In images C-E and I-O, p24mCherry construct was used. In all images, scale bars 10 µm. (9.89 MB TIF) [file pone.0005093.s003.tif]

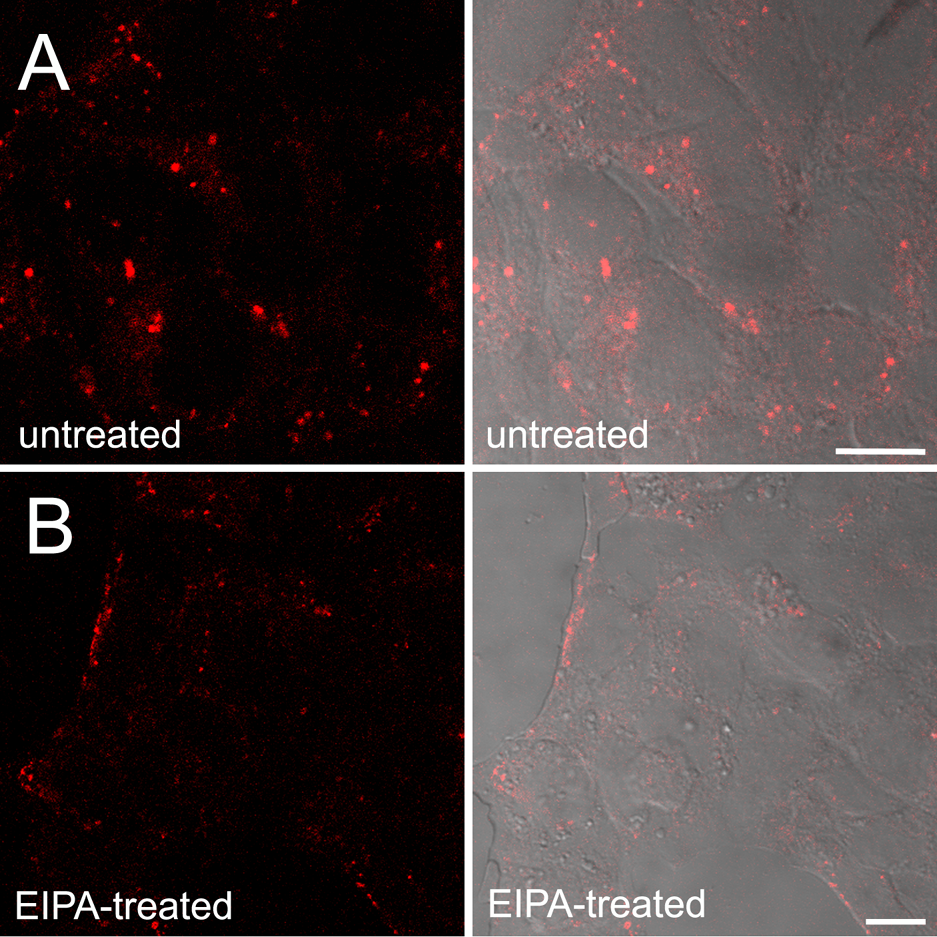

Supplement: Figure S4 — The effect of EIPA on fluid-phase entry. The efficacy of EIPA was tested by internalizing TRITC-De for 30 min in untreated (A) or EIPA-treated (0.1 mM) 293 cells (B). A representative group of cells are shown with (right) or without (left) DIC image merged with TRITC-De. Scale bars, 10 µm. (2.63 MB TIF) [file pone.0005093.s004.tif]

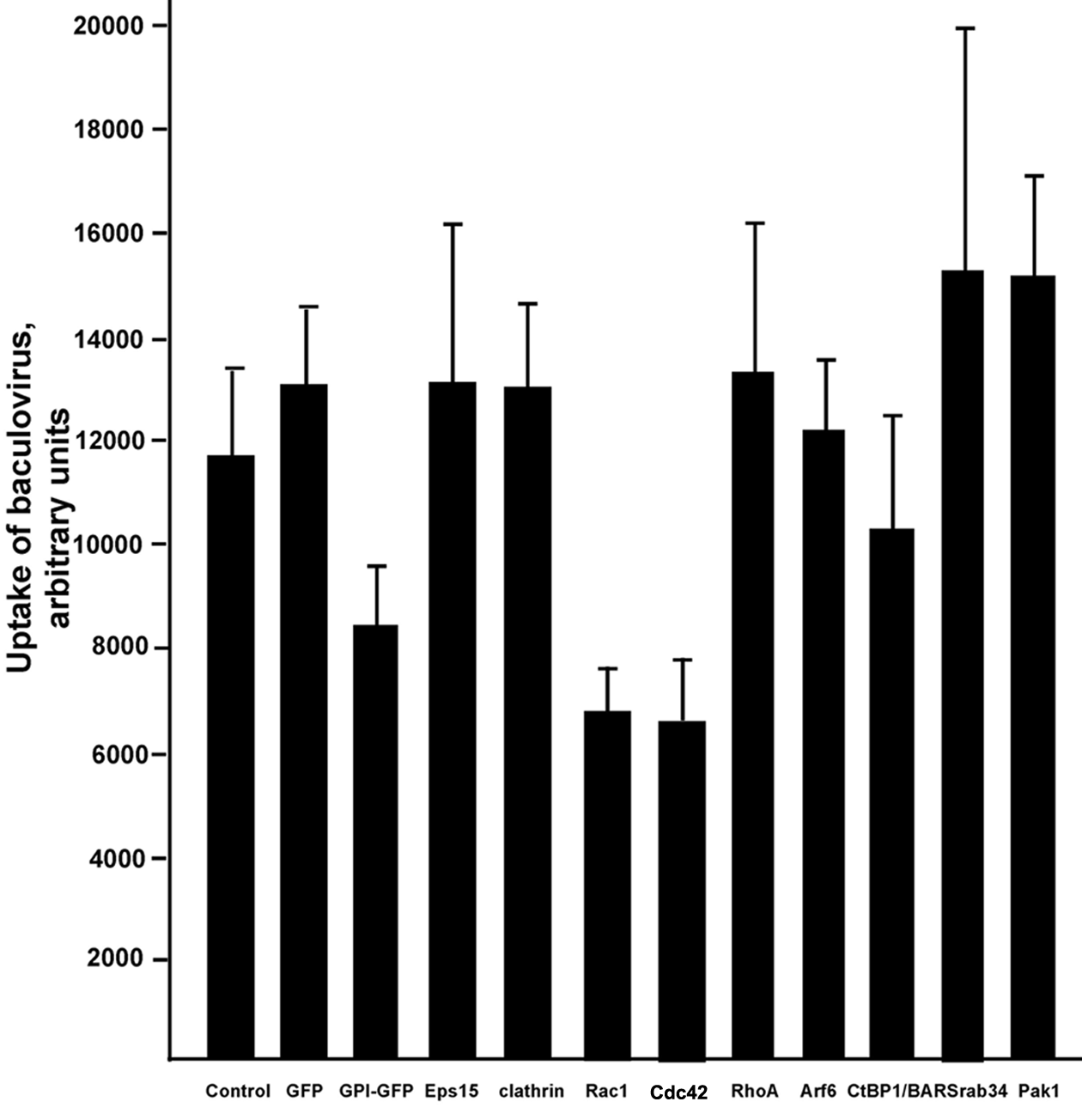

Supplement: Figure S5 — Putative effects on baculovirus entry due to expression of various endocytosis regulating WT constructs. Various plasmid constructs were transfected into 293 cells for 48 hours prior to baculovirus (p24mCherry, 200 MOI) uptake for 30 min. The plasma membrane-bound virus was extensively washed before the PFA fixation of the. Transfected cells (n = 300–400 cells) were scanned by confocal microscopy from three separate samples and analyzed for their p24mCherry fluorescence intensity. The results are shown as mean values ± SE. Untransfected cells (control) and cells expressing plain EGFP (GFP) were as controls. (1.64 MB TIF) [file pone.0005093.s005.tif]
